# When more is better

**DOI:** 10.1186/cc13791

**Published:** 2014-03-25

**Authors:** Claude Pichard

**Affiliations:** 1 Clinical Nutrition, Geneva University Hospital, Rue Gabrielle-Perret-Gentil 4, 1211 Geneva, Switzerland

## Abstract

Nutrition support of critically ill patients with sepsis is one of the most debated issues among intensivists. The latest international sepsis guidelines recommend the prescription of a low volume of feeds through gastric or intestinal enteral nutrition (EN) for 7 days after admission to the ICU. The data to support such recommendations are scarce, and large trials are needed to clarify this issue. As reported in the previous issue of *Critical Care*, Elke and colleagues have revisited a database containing 13,630 ICU patients, of whom 2,270 met four inclusion criteria: sepsis or pneumonia, ICU stay of at least 3 days, mechanical ventilation within 48 hours after ICU admission, and exclusive EN. The goal of the authors was to assess the impact of various levels of energy and protein administration on mortality at 60 days after ICU admission and on the duration of mechanical ventilation. They found that standard levels of energy and protein recommended by international guidelines for patients in the ICU do also apply to patients with sepsis in the ICU. This is an important finding, which contradicts the current recommendations and beliefs for this subgroup of patients in the ICU and gives a strong rationale for launching a large prospective randomized trial.

## 

The study by Elke and colleagues [1] in the previous issue of *Critical Care* has the great merit of dispelling a general belief that critically ill patients with sepsis should receive no feeding at all, no enteral nutrition (EN), or only minimal EN during the first week in the ICU. It also suggests that the latest recommendations should be revised.

The general recommendation for patients in the ICU is to start EN within 24 to 48 hours in order to meet the caloric goal (that is, 25 kcal/kg body weight per day) by the end of day 3 after admission [2]. The rationale for this concept is to prevent a progressive energy deficit, repeatedly associated with complications and high mortality. Unfortunately, the latest studies of the impact of EN on clinical outcome have been inconclusive. These studies suffered from methodological flaws and included a heterogeneous population of ICU patients, including those with sepsis. Nevertheless, on the basis of these works, the Surviving Sepsis Campaign Committee has released updated guidelines recommending that the prescription of EN be limited to a low volume of feeds, practically corresponding to an intentional underfeeding of sepsis patients for the 7 days after the ICU admission [3].

Elke and colleagues have formed the hypothesis that these contradictory recommendations were due mostly to an absence of robust data concerning patients with sepsis. The authors have revisited a large international database containing 13,630 ICU patients, of whom 2,270 qualified as patients with sepsis (that is, proven sepsis or pneumonia) and also met the following criteria: sepsis or pneumonia, ICU stay of at least 3 days, on mechanical ventilation within 48 hours after ICU admission, and on exclusive EN. The daily amounts of protein and energy received by the patients were then correlated to the duration on mechanical ventilation and to the 60-day mortality. The most striking results are summarized in Table 3 by Elke et al, 2014 [1]: an increase of 1,000 kcal/day was associated with a 40% reduction of the 60-day mortality and a 3-day gain of ventilator-free days. Similarly impressive improvements in outcome were observed when an additional 30 g protein/day was delivered. The authors did logically conclude that patients with sepsis should also be fed according to the general recommendations for patients in the ICU.

## Should we trust these conclusions?

Yes, for the following reasons: (a) the data were prospectively recorded in 737 ICUs in 33 countries and this reduces the impact of local medical practice and optimizes the external validity of the studied patient population, (b) the respective contribution of various specific medical conditions is minimized by the large number of patients, (c) the inclusion and exclusion criteria were easy to apply and this allows a clear typology of the patients enrolled in the study, (d) the selected outcomes are clinically relevant, and (e) the statistical analysis was carried out by one of the reference teams.

## Are there methodological weaknesses to be highlighted?

Yes, such as the following. First, the energy needs were calculated mostly by predictive equations and rarely (13%) measured by indirect calorimetry. As mentioned by the authors themselves and in recent works [4,5], ‘critically ill patients are characterized by marked variations in energy requirements’, and this is especially true of patients with sepsis. Given that they have a liver vulnerability to overfeeding, measurement of energy needs should be (come) the gold standard. Second, the enrollment was limited to patients with medical conditions and this precludes direct applications to surgical patients. Third, the composition of the nutrition products is not precisely described, nor is the administration of micronutrients. Some immune-enhancing compounds may have influenced the clinical outcome [6,7]. Fourth, the intolerance to EN, mostly vomiting and diarrhea is not documented [8] and may have influenced the prescription of EN. Also, the duration of mechanical ventilation is reported, but the contribution of non-invasive ventilation to the total ventilation duration is not described. Finally, the 60-day mortality is an irrefutable parameter but is more the reflection of the medical conditions and the combined actions of all the therapies (sort of a black box!) than of the specific action of the nutritional therapy.

In summary, this excellent study highlights the positive contribution of the nutritional therapy to the clinical outcome of patients with sepsis in the ICU. It suggests that general guidelines for patients in the ICU also apply to the patients with sepsis. A comprehensive prospective randomized trial is needed to draw a firm conclusion. This study should use the same clinical endpoints and include the determination of energy target by indirect calorimetry in order to allow an individualized optimal prescription of energy [7,9].

## Abbreviations

EN: Enteral nutrition.

## Competing interests

The author declares that he has no competing interests.
